# Evaluating the efficacy and cardiotoxicity of EGFR-TKI AC0010 with a novel multifunctional biosensor

**DOI:** 10.1038/s41378-023-00493-4

**Published:** 2023-05-10

**Authors:** Deming Jiang, Xinwei Wei, Yuxuan Zhu, Yong Qiu, Xin Liu, Liubing Kong, Fengheng Li, Jingwen Liu, Liujing Zhuang, Hao Wan, Kejing Ying, Ping Wang

**Affiliations:** 1 Biosensor National Special Laboratory, Key Laboratory for Biomedical Engineering of Education Ministry, Department of Biomedical Engineering, Zhejiang University, Hangzhou, Zhejiang, 310027 China; 2 Innovation Center for Smart Medical Technologies & Devices, Binjiang Institute of Zhejiang University, Zhejiang, 310053 China; 3 Cancer Center, Zhejiang University, Hangzhou, Zhejiang, 310058 China; 4 College of Pharmaceutical Sciences, Zhejiang University, Hangzhou, Zhejiang, 310058 China; 5 Department of Gastroenterology, Zhejiang University School of Medicine Second Affiliated Hospital, Hangzhou, Zhejiang, 310009 China; 6 Department of Respiratory and Critical Medicine, Sir Run Run Shaw Hospital, School of Medicine, Zhejiang University, Hangzhou, China; 7 State Key Laboratory for Sensor Technology, Chinese Academy of Sciences, Shanghai, 200050 China

**Keywords:** Chemistry, Engineering

## Abstract

Non-small cell lung cancer (NSCLC) is a leading cause of cancer mortality worldwide. Although epidermal growth factor receptor tyrosine kinase inhibitors (EGFR-TKIs) have dramatically improved the life expectancy of patients with NSCLC, concerns about TKI-induced cardiotoxicities have increased. AC0010, a novel third-generation TKI, was developed to overcome drug resistance induced by EGFR-T790M mutation. However, the cardiotoxicity of AC0010 remains unclear. To evaluate the efficacy and cardiotoxicity of AC0010, we designed a novel multifunctional biosensor by integrating microelectrodes (MEs) and interdigital electrodes (IDEs) to comprehensively evaluate cell viability, electrophysiological activity, and morphological changes (beating of cardiomyocytes). The multifunctional biosensor can monitor AC0010-induced NSCLC inhibition and cardiotoxicity in a quantitative, label-free, noninvasive, and real-time manner. AC0010 was found to significantly inhibit NCI-H1975 (EGFR-L858R/T790M mutation), while weak inhibition was found for A549 (wild-type EGFR). Negligible inhibition was found in the viabilities of HFF-1 (normal fibroblasts) and cardiomyocytes. With the multifunctional biosensor, we found that 10 μM AC0010 significantly affected the extracellular field potential (EFP) and mechanical beating of cardiomyocytes. The amplitude of EFP continuously decreased after AC0010 treatment, while the interval decreased first and then increased. We analyzed the change in the systole time (ST) and diastole time (DT) within a beating interval and found that the DT and DT/beating interval rate decreased within 1 h after AC0010 treatment. This result probably indicated that the relaxation of cardiomyocytes was insufficient, which may further aggravate the dysfunction. Here, we found that AC0010 significantly inhibited EGFR-mutant NSCLC cells and impaired cardiomyocyte function at low concentrations (10 μM). This is the first study in which the risk of AC0010-induced cardiotoxicity was evaluated. In addition, novel multifunctional biosensors can comprehensively evaluate the antitumor efficacy and cardiotoxicity of drugs and candidate compounds.

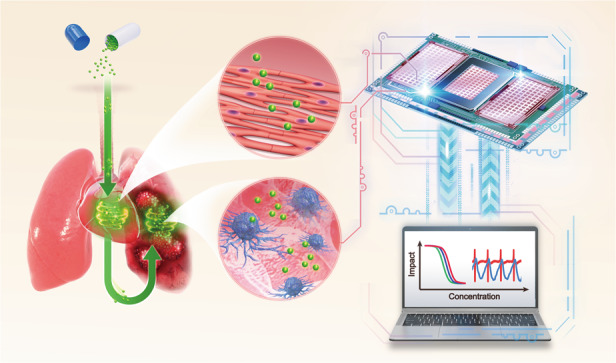

## Introduction

Non-small cell lung cancer (NSCLC) is the major cause of cancer-related deaths worldwide in both men and women^1^. Epidermal growth factor receptor (EGFR) mutations have been identified in approximately 30% to 40% of patients with NSCLC in Asia, which is much higher than non-Asian patients^2,3^. The EGFR family is a family of receptor tyrosine kinases that play critical roles in signal transduction and activating multiple cytoplasmic signaling pathways that can promote cancer cell survival, proliferation, and migration. Small molecule tyrosine kinase inhibitors (TKIs) have been suggested proven to significantly improve tumor inhibition and progression-free survival (PFS) for patients harboring this mutation^4,5^. A relevant EGFR mutation-positive NSCLC patient benefits from TKI therapy, including gefitinib, erlotinib, icotinib, and afatinib^6^. However, several secondary mutations were reported to confer acquired resistance to EGFR-TKIs, including T790M, D761Y, L747S, and T854A^7^. Among them, T790M was found in approximately half of the TKI-treated patients. Second-generation TKIs, such as dacomitinib and afatinib, were developed to overcome acquired resistance^8,9^; however, the overall survival was negligibly improved in clinical trials^9^. AC0010, a novel irreversible EGFR-TKI cored with pyrrolopyrimidine, was developed to overcome T790M-induced resistance in NSCLC patients^10^. The first phase studied showed that AC0010 was well tolerated and effective in NSCLC patients who had acquired resistance from previous TKI treatment^11^.

Although TKIs showed high potential for NSCLC patients, severe cardiotoxicities were linked to some TKIs, including arrhythmias, reduced left ventricular ejection fraction, myocardial infarction, or heart failure^12,13^. Gefitinib, a widely used TKI, was observed to cause recurrent myocardial infarction in patients^14^. Studies have demonstrated that abnormal oxidative stress pathways^15^ and the PTEN/AKT/FoxO3a pathway^16^ are probably involved in gefitinib-induced cardiotoxicities. Sharma et al. reported a cardiac safety index to widely assess TKI-induced cardiotoxicities^17^. Vemurafenib, sorafenib, regorafenib, vandetanib, crizotinib, and nilotinib were found to cause significant cardiotoxicities in applications. As a new third-generation tyrosine kinase inhibitor, AC0010 provides an improved life expectancy for patients with TKI-resistant NSCLC. However, it is unclear whether AC0010 results in cardiotoxicities during clinical application.

In drug discovery programs, the electrophysiological patch clamp assay is the “gold standard” to evaluate compound-induced ether-a-go-go related gene (hERG) blockade, which is closely related to QT prolongation^18^. However, a number of potential errors in operation can lead to misinterpretation of results. In recent years, biosensor technology has emerged as an effective approach to assess cell survival and physiological activities and has been widely used in preclinical studies and drug screening^19,20^.

**Fig. 1:**
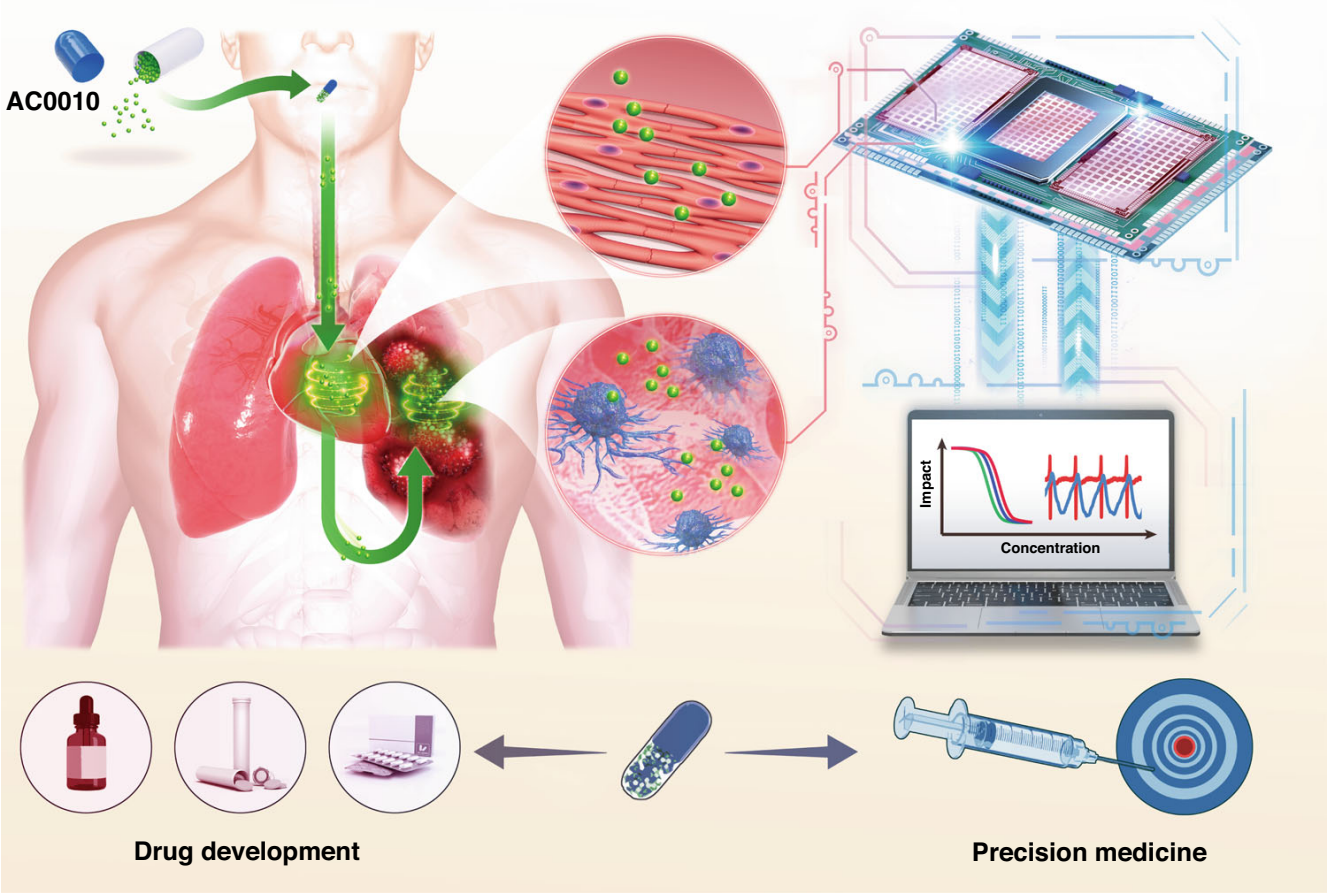
Scheme showing the evaluation of AC0010-induced NSCLC inhibition and cardiotoxicity using the multifunctional biosensor. Cardiotoxicity is a common side effect of oral targeted drugs. A simplified heart chip and a lung cancer chip were constructed by seeding cardiomyocytes and NSCLC cells on the sensor chip. By converting cell activity and physiological function into electrical signals that are easy to read and analyze, the novel multifunctional biosensor can help researchers evaluate anti-lung cancer efficacy and cardiotoxicity in a noninvasive, real-time, and precise manner. Both new drug development and precision treatment can benefit from the development of multifunctional biosensors

In previous work, we reported a dual-functional biosensor to assess Taxol- and vinblastine-induced cancer cell inhibition and cardiotoxicity^21,22^. The biosensor can measure cell viability and electrophysiological activity simultaneously. However, other important characteristics of cardiomyocytes, such as mechanical contractile signals, are ignored, which is indispensable for evaluating cardiotoxicity. Thus, we built a novel multifunctional biosensor that is composed of microelectrodes (MEs) and interdigital electrodes (IDEs). Through the MEs, the extracellular field potential of cardiomyocytes is efficiently recorded. Electrical cell-substrate impedance sensing (ECIS) based on IDEs was developed for cell viability assays and drug toxicity screening in our previous works^20,23^. By increasing the sampling frequency to 2 ms/point, the IDEs could record cardiomyocyte mechanical beating based on impedance signals. We aimed to simultaneously evaluate AC0010-induced cancer cell inhibition and cardiotoxicities with the novel multifunctional biosensor (Fig. 1). Notably, this is the first work to explore AC0010-induced cardiotoxicities, which can provide instructive information for future clinical trials and applications.

## Results

### Assessment of AC0010-induced anti-NSCLC efficacy with the multifunctional biosensor

AC0010 has demonstrated the ability to strongly inhibit the EGFR T790M mutation while overlooking wild-type EGFR. The acrylamide groups on AC0010 are predicted to react with Cys797 from the EGFR T790M mutation based on molecular modeling^10^. Compared to prymindine-based compounds, such as rociletinib, WZ4002, and osimertinib, AC0010 showed better selectivity and affinity for EGFR T790M mutations^10^. To further evaluate the selectivity and anticancer efficacy of AC0010, we used NCI-H1975 (EGFR L858R/T790M NSCLC cells), A549 (wild-type EGFR NSCLC cells), and HFF-1 (fibroblasts) cells. In the present study, three cell lines (NCI-H1975, A549, and HFF-1) were cultured on the biosensor and treated with AC0010 at concentrations ranging from 0 μM to 100 μM.

**Fig. 2:**
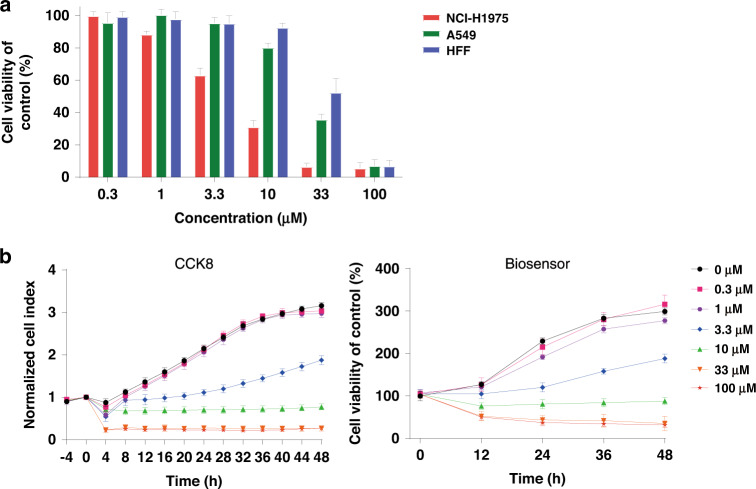
AC0010-induced inhibition evaluated with the multifunctional biosensor. **a** Cell viability of NCI-H1975, A549, and HFF cells after treatment with AC0010 for 48 h. **b** Cell viability of NCI-H1975 cells after treatment with AC0010 was followed with the CCK8 assay and the multifunctional biosensor

Electrical impedance data, which are recorded by the multifunctional biosensor, are commonly quantified to the cell index (CI), a ratio of the impedance change (ΔZ) to background impedance (Z_0_), to reflect cell growth and death^24^. After 48 h of treatment, the CI values were calculated to evaluate the cell viability. As shown in Fig. 2a, AC0010 inhibited NCI-H1975 (EGFR L858R/T790M) in a dose-dependent manner with an IC50 value of 5.15 μM. A poor response was found for A549 cells, and no inhibition was observed for HFF cells (Fig. 2a). The results demonstrated that AC0010 exhibits a stronger inhibitory effect on EGFR-mutant NSCLC cells harboring the T790M mutation and overlooked EGFR-wild-type NSCLC cells and normal cells. As the T790M mutation is the major cause of TKI therapy-induced resistance, AC0010 is crucial for patients with recurrent tumors.

Then, we performed multiple CCK8 assays to verify the reliability of our multifunctional biosensor (Fig. 2b). NCI-H1975 cells were seeded on 96-well plates at the same density as the biosensor assays. Multiple CCK8 assays were performed accordingly, while cell viabilities before and after treatment were calculated and plotted. The plots performed by the two methods were similar, while a much higher abundance was found with the biosensor. The sharp decreases in CI values were probably due to AC0010 addition-induced changes in cell morphology and the medium environment^25^. The results demonstrated that the multifunctional biosensor is appropriate for evaluating anticancer efficacies. Under the same experimental workflow, the multifunctional biosensor strategy can provide more time abundance data than that of traditional test methods, which makes it a good supplementary or alternative for life science research.

### Assessment of the AC0010-induced effect on cardiomyocyte viability with the multifunctional biosensor

**Fig. 3:**
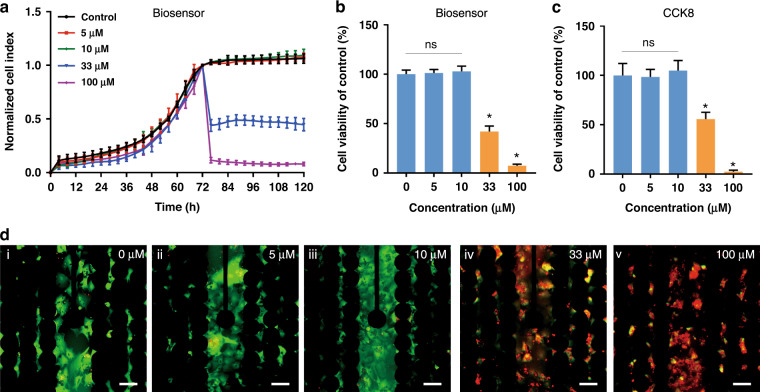
AC0010 of effective concentration for NSCLC is tolerable for cardiomyocytes. **a** CI curves normalized to the time of adding AC0010. **b** Cell viability calculated with CI values obtained from the multifunctional biosensor after 48 h of treatment. **c** Cell viability calculated by CCK8 assay after 48 h of treatment. **d** Live-dead assay results after treatment with AC0010 at 0 μM (i), 5 μM (ii), 10 μM (iii), 33 μM (iv), and 100 μM (v) on the multifunctional biosensor. Error bars represent the mean ± SD from three replicates, *p < 0.05. Bar = 100 μm

Then, we used the multifunctional biosensor to determine whether AC0010 is toxic to cardiomyocyte viability. Primary cardiomyocytes were extracted from neonatal rats and purified by two adhesion cycles. Cardiomyocytes were seeded at a density of 25,000 cells per well. As shown in Supplementary Fig. S1, immunofluorescence results confirmed that cardiomyocytes were stained with the cardiomyocyte-specific markers cTnI (red) and cardiac muscle α-actin (green). Images showed that cardiomyocytes clustered and beat spontaneously on the electrodes (Supplementary Fig. S2), as previously reported by other researchers^26,27^. CI values were found to increase in the early stage and then remained stable (Fig. 3a). Primary cardiomyocytes adhered and spread on the chip, resulting in an increase in CI values. After approximately 72 h, the cardiomyocytes reached a steady state, and the CI values remained stable because the cardiomyocytes could not proliferate^28^. As 10 μM AC0010 induces significant inhibition of the NCI-H1975 cell line, we treated the cardiomyocytes with AC0010 at concentrations ranging from 5 μM to 100 μM. According to the growth curves, no significant differences were found in viability after treating the cardiomyocytes with 0, 5, and 10 μM AC0010 (Fig. 3a, b). However, significant toxicity to viability was observed at higher concentrations (33 μM and 100 μM).

To verify the results of the multifunctional biosensor, a CCK8 assay and live/dead staining were conducted. As shown in Fig. 3c, the CCK8 assay proved that when the concentrations were equal to or lower than 10 μM, no inhibition was observed. Fluorescence images of live/dead staining were also consistent with the results of our multifunctional biosensor (Fig. 3d). After treatment with AC0010 at 0 μM, 5 μM, and 10 μM, almost no dead cells (stained red) were observed. With an increasing concentration of AC0010, there were progressive increases in cell death. Overall, 10 μM AC0010 induced strong inhibition of NCI-H1975 without obvious toxicity to cardiomyocyte viability. As the primary objective was to validate the application of the multifunctional biosensor for cardiotoxicity evaluation, we selected a concentration of 10 μM for subsequent experiments.

### Recording electrophysiological and beating activity of cardiomyocytes with the multifunctional biosensor

**Fig. 4:**
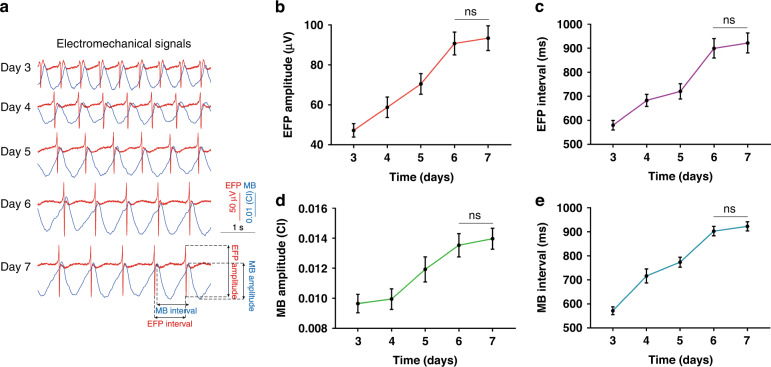
The maturation process of isolated cardiomyocytes. **a** Typical extracellular field potential (EFP) and mechanical beating (MB) signals of cardiomyocytes from Day 3 to Day 7; statistics of (**b**) EFP amplitude, (**c**) EFP interval, (**d**) MB amplitude, and (**e**) MB interval on different days

We recorded the extracellular field potential (EFP) and mechanical beating (MB) of cardiomyocytes with the multifunctional biosensor. As shown in Fig. 4a, EFP signals and MB signals gradually increased during the process of cultivation, which was probably due to cell maturation. During culture, the EFP amplitude increased from 47.25 ± 3.389 μV on Day 3 to 93.45 ± 6.178 μV on Day 7 (Fig. 4b). In addition, the EFP interval increased from 579.8 ± 19.85 ms on Day 3 to 922.1 ± 41.31 ms on Day 7 (Fig. 4c). The amplitude and interval of MB increased consistently with the EFP from 0.0097 ± 0.00062 CI on Day 3 to 0.0140 ± 0.00070 CI on Day 7 and from 571.7 ± 16.52 ms on Day 3 to 923.0 ± 19.07 ms on Day 7 (Fig. 4d, e). The EFP firing and mechanical beating occurred consistently and regularly. Cardiomyocytes seemed to mature after 6 days of culture in vitro because there was no significant difference between Day 6 and Day 7. Thus, AC0010 addition occurred after 6 days of culture.

### Evaluation of AC0010-induced effects on the electrophysiological activity of cardiomyocytes with the multifunctional biosensor

**Fig. 5:**
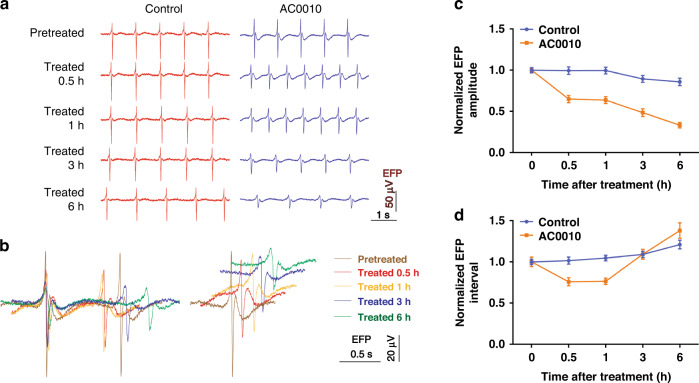
AC0010-induced changes in EFP signals. **a** Typical EFP signals of cardiomyocytes with and without 10 μM AC0010 treatment; **b** Superposition of EFP waveforms of cardiomyocytes before and after treatment; Normalized amplitudes (**c**) and intervals (**d**) of EFP with and without 10 μM AC0010 treatment

Representative EFP signals with and without exposure to 10 μM AC0010 are shown in Fig. 5a. Obvious changes in the waveform were recorded after AC0010 treatment with the multifunctional biosensor. Compared with that of the control group, the EFP amplitude decreased immediately and continuously, while the EFP interval decreased first and then increased. By overlaying waveforms of different time points, significant differences were observed (Fig. 5b).

The amplitudes and intervals of EFP were analyzed and normalized by the values before treatment (Fig. 5c, d). For the control group, slight changes were found after the medium was renewed. The amplitudes decreased from 90.26 ± 2.82 μV to 89.80 ± 4.15 μV (0.5 h), 89.88 ± 3.60 μV (1 h), 80.70 ± 3.73 μV (3 h), and 77.50 ± 4.06 μV (6 h). Furthermore, the intervals increased from 925.8 ± 30.62 ms to 940.6 ± 39.29 ms (0.5 h), 969.7 ± 31.83 ms (1 h), 1010.0 ± 34.91 ms (3 h), and 1121.0 ± 49.16 ms (6 h). Significant changes were found after 3 h or longer, while fresh medium change can restore the EFP activities (data not shown), probably due to the depletion of nutrients, as much energy is consumed by beating.

For the AC0010 group, significant changes were found immediately after AC0010 treatment. The amplitudes decreased quickly from 93.29 ± 2.49 μV to 60.57 ± 4.08 μV (0.5 h), 59.41 ± 3.84 μV (1 h), 45.24 ± 4.52 μV (3 h) and 30.93 ± 3.15 μV (6 h). After 6 h of treatment, the amplitude decreased significantly to 33.16 ± 3.38%. Unlike the amplitudes, the intervals decreased from 908.8 ± 52.96 ms to 689.3 ± 44.38 ms (0.5 h) and 694.1 ± 35.67 ms (1 h) first and then increased to 993.0 ± 55.05 ms (3 h) and 1254.9 ± 85.82 ms (6 h). It seems that AC0010 can interfere with the frequency of the active potential.

### Evaluating the AC0010-induced effects on the beating of cardiomyocytes with the multifunctional biosensor

**Fig. 6:**
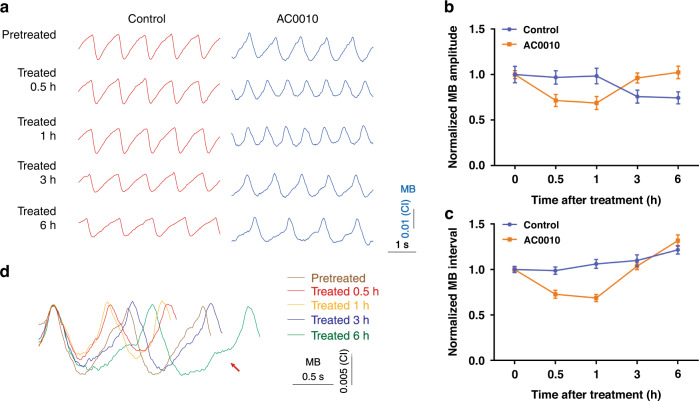
AC0010-induced changes in MB signals. **a** Typical MB signals of cardiomyocytes with and without 10 μM AC0010 treatment; Normalized amplitudes (**b**) and intervals (**c**) of MB with and without 10 μM AC0010 treatment; **d** Superposition of MB waveforms of cardiomyocytes before and after treatment

With the multifunctional biosensor, we also evaluated whether 10 μM AC0010 impairs the beating function of cardiomyocytes. Representative MB signals before and after exposure to 10 μM AC0010 are shown in Fig. 6a. Similar to the EFP signals, a slight increase in intervals and a decrease in amplitudes were recorded over time for the control group. MB amplitudes decreased from 0.0137 ± 0.00124 CI to 0.0133 ± 0.00103 CI (0.5 h), 0.0135 ± 0.00117 CI (1 h), 0.0104 ± 0.00097 CI (3 h), and 0.0102 ± 0.00090 CI (6 h), while the intervals increased from 938.1 ± 31.41 ms to 926.7 ± 38.14 ms (0.5 h), 996.3 ± 45.66 ms (1 h), 1032.0 ± 58.89 ms (3 h), and 1142.0 ± 43.72 ms (6 h).

After 10 μM AC0010 treatment, significant differences in beating waveforms were recorded. The changes were similar to the EFP signals. Obviously decreased amplitudes and intervals were observed within 1 h, causing more frequent but incomplete beatings. With prolonged exposure time, the amplitudes and intervals increased (Fig. 6b, c). After exposure to AC0010, the amplitudes of MB dropped from 0.0122 ± 0.00054 CI to 0.0087 ± 0.00081 CI (0.5 h), 0.0084 ± 0.00087 CI (1 h) first and then increased to 0.0117 ± 0.00067 CI (3 h) and 0.0125 ± 0.00084 CI (6 h). Similarly, the intervals of MB first increased from 968.7 ± 35.1 ms (3 h) to 1232.0 ± 59.45 ms (6 h). By overlaying waveforms of different time points, significant differences were observed (Fig. 6d).

### Evaluation of AC0010-induced effects on the contraction and relaxation function of cardiomyocytes with the multifunctional biosensor

**Fig. 7:**
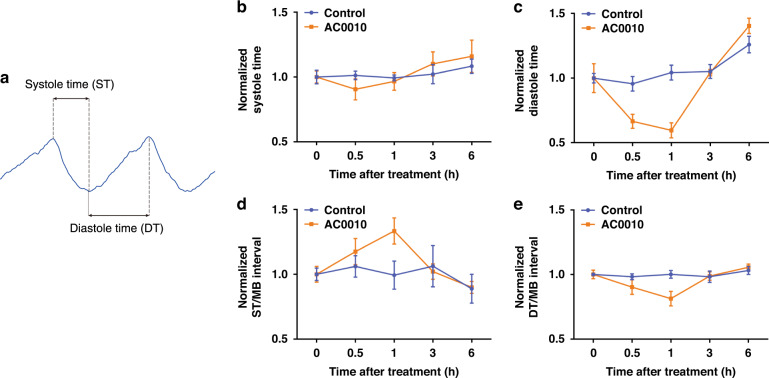
AC0010 causes abnormalities in the contraction and relaxation function of cardiomyocytes. **a** Rule to separate an MB interval into a systole time (ST) and a diastole time (DT). Normalized ST (**b**), DT (**c**), ST/MB interval (**d**), and DT/MB interval (**e**) of cardiomyocytes with and without 10 μM AC0010 treatment

To further investigate AC0010-induced effects on beating function, we separated the MB interval into the systole time (ST) and diastole time (DT), as shown in Fig. 7a. Within 1 h after AC0010 treatment, both ST and DT dropped significantly due to the more frequent EFP firing, while DT exhibited a larger decrease (Fig. 7b, c). By analyzing the ratio of ST and DT to the MB interval (Fig. 7d, e), we found that the ST/MB interval ratio significantly increased while the DT/MB interval ratio decreased. A decreased proportion of DT might be related to abnormal cardiomyocyte activities. It is well known that sufficient diastole is important for cardiomyocyte function and human health^29^. During diastole, Ca^2+^ ions are pumped to the sarcoplasmic reticulum, and cardiomyocytes return to an unstressed length and force, preparing for the next beats. Due to continued diastole deficiency, the cardiomyocyte bundle enters a state of constant stress, probably impairing structure and function.

### Visualization of AC0010-induced effects detected by the multifunctional biosensor

**Fig. 8:**
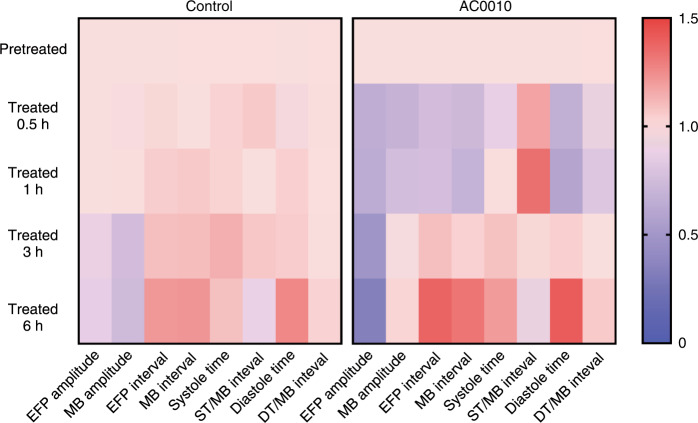
AC0010 causes an apparent influence on cardiomyocytes’ physiological activities. Heatmaps to visualize the time-induced changes and AC0010-induced changes in the parameters recorded by the multifunctional biosensor

To observe AC0010-induced effects on cardiomyocytes more intuitively, we generated a heatmap of normalized EFP amplitudes, MB amplitudes, EFP intervals, MB intervals, ST, ST/MB interval ratios, DT, and DT/MB interval ratios with and without 10 μM AC0010 treatments (Fig. 8). In the heatmap, the regions labeled red indicate upregulation, while blue indicates downregulation. In the control group, most changes occurred after 3 h and 6 h of treatment, probably because the physiological activities of cardiomyocytes consume a large amount of energy. In the AC0010 group, obvious changes were found 1 h after treatment, in which significant upregulation of the ST/MB interval ratio and downregulation of the EFP amplitude, MB amplitude, EFP interval, MB interval, DT, and DT/MB interval ratio were observed. With the heatmap, distinct differences were clearly observed after 10 μM AC0010 treatment, indicating that AC0010 induced obvious changes in the electrophysiological and beating activities of cardiomyocytes.

## Discussion

Cardiotoxicity remains a major cause of concern for antitumor drugs during preclinical and clinical development as well as postapproval withdrawal of medicines. Approximately 45% of the postapproval drugs withdrawn from the market are withdrawn due to cardiovascular system risk^30^. In this study, we provided a convenient method to test the potency and cardiovascular side effects of drug candidates in preclinical studies, which can help reduce the withdrawal rate. The multifunctional biosensor was capable of measuring cell viability, electrophysiological activity, and mechanical beating simultaneously. We proved that AC0010 caused significant antitumor activity in EGFR-T790M mutant NSCLC cells, and for the first time, we found that AC0010 might induce some adverse effects on cardiomyocytes.

Over the past decade, biosensing technologies have been developed for cardiotoxicity evaluations as novel, label-free, real-time, high throughput, and noninvasive/minimally invasive tools^31^. Biosensors are capable of detecting and recording the cell viability, electrophysiological activity, mechanical motion, and contractile force of cardiomyocytes. Compared to traditional methods, such as patch clamp^32^ and Ca^2+^ imaging^33^ for measuring electrophysiological activity, biosensors work in a more biocompatible and efficient way. Since they were proposed in 1972^34^, microelectrode arrays (MEAs) have been applied for drug-induced arrhythmia^35^ and QT prolongation^36^ screening by integrating cardiomyocytes. Our group previously used MEA to rapidly detect marine toxins^37^ and bitter and umami compounds^38^ with high sensitivity and selectivity. Electrical cell-substrate impedance sensing (ECIS) technology, introduced by Giaever and Keese^39,40^, has been widely applied for drug screening and cardiotoxicity evaluation^41–43^. In an ECIS system, cells are seeded onto interdigital electrodes (IDEs), while cell motion, deformation, division, and death change the impedance values recorded by the electrodes^44^. Many studies have been performed to investigate the anticancer efficacy of drugs with electrical impedance sensors^45^. As electrical impedance signals can reflect cell motion and deformation, electrical cell-substrate impedance sensors can monitor stimulation-induced cell responses^46^. By increasing the sampling rate, we precisely recorded electrically coupled excitation and contraction signals of cardiomyocytes^47^. In this work, we combined the advantages of MEs and IDEs to evaluate the anticancer efficacy and cardiac toxicity of AC0010 with a novel multifunctional biosensor. Compared to previous strategies, this noninvasive and in vitro drug evaluation strategy can provide comprehensive information on drugs more easily and quickly. In addition, biosensors are easily integrated into dense arrays, which provides a powerful and high-throughput detection and screening method.

In recent years, heart-on-a-chip technology has attracted increasing attention in drug screening because the technology can stimulate the structure and function of the human body in vivo. Heart-on-a-chip systems are mostly built on a microfluidic chip; thus, low volumes are needed and rapid evaluations can be achieved for high-throughput screening. By integrating biosensing technology, a composite heart-on-a-chip platform shows significantly enhanced detection sensitivity for dynamically displaying cardiomyocytes. For example, Shao et al. introduced an anisotropic structural color graphene film to detect the autonomous beating of cardiomyocytes and applied it in a heart-on-a-chip platform for drug screening^48^. On the flexible graphene film, attached cardiomyocytes can cyclically bend the film during beating and show structural color. Biocompatible hydrogel-based structural color films can also function as optical sensors to monitor cardiomyocyte behavior^49^. Compared with optical sensors, bioelectronic sensors (such as our multifunctional biosensor) show higher sensitivity and can be reused^50^. In a follow-up study, we will further explore the integration of bioelectronics and microfluidic channels, which is extremely valuable in the field of drug screening and other biomedical applications.

However, the multifunctional biosensor was based on MEs and IDEs and was more compatible with plate-cultured cells. To combine with more complex and biomimetic cardiac models, including 3D cardiac structures^51^, spheroids^52^, and organoids^53^, biomaterial-based scaffolds could be used. For example, Wei et al. combined porous scaffold-based engineered cardiac tissue with microelectrode arrays (MEAs) for advanced pharmaceutical studies^54^. Bioengineered cardiac tissues were compatible with the multifunctional biosensor, which can be used to expand the applications in pharmaceutical studies and preclinical studies.

With the multifunctional biosensor, we revealed for the first time that AC0010 might induce side effects on cardiomyocytes, affecting electrophysiological and beating functions. This study demonstrated that 10 μM AC0010 accelerates the frequency of action potential within 1 h and consumes much ATP, resulting in subsequent recession of EFP and MB signals. In addition, we revealed that AC0010 reduced the diastole time during the beat interval with more frequent action potential firing. With the surge of anticancer drugs, an emerging concern is the risk for drug-induced ventricular arrhythmias and sudden death. A retrospective study including 2301 reports indicated that kinase inhibitors caused the most drug-induced long QT syndrome^55^, probably due to the inevitable off-target effects^56^.

Although we have provided a powerful tool for evaluating the cardiac toxicity of AC0010, the underlying mechanism remains ambiguous and is outside the scope of this work. Identifying the underlying mechanism can contribute to the development of new targeted drugs. For a majority of TKIs, there is a wide gap in our knowledge regarding the types and risk of cardiotoxicity^57^. Many multitargeted TKIs result in applications in more types of cancer, but with this comes a higher risk of cardiac toxicity. The inhibition of ‘bystander’ targets is probably involved in cardiomyocyte survival^58^. For example, vascular endothelial growth factor receptor 2 (VEGFR2)/platelet-derived growth factor receptor (PDGFR)-inhibiting TKIs were reported to cause a compensatory increase in insulin and insulin-like growth factor (IGF) signaling in induced pluripotent stem cell-derived cardiomyocytes (hiPSC-CMs)^17^. It has been revealed that drug-induced cardiotoxicity arises from the accumulation of oxidative stress, disruption of calcium homeostasis, and abnormalities in the transcriptome and proteome^59^. Increased reactive oxygen species (ROS) and cytomembrane permeability damage (increased lactate dehydrogenase release) are commonly identified in TKI-damaged cardiomyocytes^60^.

As AC0010 is newly developed, much future work should be carried out. Improving detecting instruments and investigating underlying mechanisms are mutually beneficial strategies, which can contribute to the development of new therapies and save people’s lives and health.

## Conclusion

Adverse effects in anticancer therapy and new drug development are common and difficult to overcome. AC0010, a new generation of TKIs, showed impressive inhibition of EGFR T790M-mutant NSCLC cell lines in vitro. However, TKI-associated cardiotoxicities cause widespread concern in clinical application. As no study has reported a relationship between AC0010 and cardiotoxicities, we investigated this subject with our novel multifunctional biosensor. The multifunctional biosensor, consisting of microelectrodes (MEs) and interdigital electrodes (IDEs), is capable of simultaneously recording viability, mechanical beating, and electrophysiological signals. Based on the biosensor results, we found that 10 μM AC0010 induced strong inhibition of EGFR-T790M mutant NSCLC cell lines without affecting cardiomyocyte viability. In addition, we used the multifunctional biosensor to record cardiomyocyte mechanical beating and electrophysiological signals before and after AC0010 treatment. The results showed that 10 μM AC0010 significantly increased the frequency while reducing the amplitude of the extracellular field potential and mechanical beating within 1 h. When time was prolonged, cardiomyocyte action potential and beating were repressed in turn. For the first time, we revealed the potential risk of AC0010 in the cardiovascular system, and more in-depth studies are needed in future trials. It is worth noting that our multifunctional biosensor can comprehensively evaluate drug-induced anticancer efficacy and cardiotoxicities in a real-time and noninvasive manner, showing promising potential in pharmaceutical research and new drug development.

## Materials and methods

### Biosensor fabrication

**Fig. 9:**
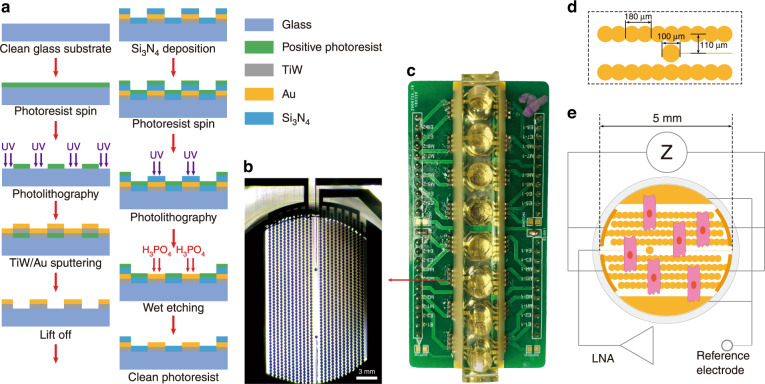
Construction of the multifunctional biosensor. **a** Fabrication procedures of the microelectrodes (MEs) and interdigital electrodes (IDEs). **b** Optical image of the electrodes. **c** Picture of the fabricated biosensor chip; **d** Partial enlarged detail of the designed electrodes. **e** Working principle of the multifunctional cell-based biosensor (LNA: low-noise amplifier)

A 4-inch quartz glass wafer (Corning, USA) was used to fabricate interdigital electrodes (IDEs) and microelectrodes (MEs). As shown in Fig. 9a, we patterned 10 nm Ti/100 nm Au using the positive photoresist Microposit S1813 (Shipley, USA). The negative photoresist SU-8 2002 (Microchem, USA) was used to insulate the leads of IDEs and MEs. Two 100 μm diameter MEs were patterned in the middle area, and the center-to-center distance between the two MEs was 3 mm. Two circle-on-line interdigitated branches with a diameter of 90 μm were patterned in two side areas. The center distance of adjacent interdigitated branches was 110 μm, and the distance between the ME and IDE was 50 μm. Two reference electrodes were designed at the edge of the IDEs (Fig. 9b). Finally, a polymethyl methacrylate (PMMA) chamber (5 mm diameter) was integrated for cell culture (Fig. 9c).

### Working principle of the multifunctional cell-based biosensor

The MEs and IDEs of the multifunctional biosensor were utilized to record the extracellular field potential (EFP) and cell impedance, respectively (Fig. 9d, e). Na^+^, K^+^, and Ca^2+^ flow through the cardiomyocyte membrane and trigger the action potential. Cardiomyocytes generate transient transmembrane potential and ionic current in rhythm. The potential reconstructs the charge distribution at the electrode-electrolyte-cell interface and polarizes the MEs. We used a low-noise amplifier (LNA) to amplify the changed voltage, which was recorded as EFP. Cell impedance recorded by the IDEs can monitor cell viability and mechanical beating. According to a previous study, sinusoidal voltage (amplitude of 30 mV and frequency of 10 kHz) was applied to the IDEs, and an ion current was generated, converted to impedance signals, and recorded^47^. Due to the slight distinction of electrodes and the combination of cells and electrodes, the recorded background impedance values are different. Adhesion, growth, proliferation, migration, death, and morphological changes in cells affect the ion current and impedance values. Thus, the cell index (CI) was calculated as the ratio of the cell impedance change ΔZ to background impedance Z_0_ (CI = ΔZ/Z_0_) to reflect cell behavior in real time^61^. When the sampling frequency was increased to 2 ms/point, the multifunctional biosensor could record contraction and reflaxation-induced morphological changes in cardiomyocytes.

### Cell source and cultivation

NSCLC cell lines NCI-H1975 and A549 and human foreskin fibroblasts (HFF-1) were purchased from ATCC. NCI-H1975 and A549 cells were cultured in RPMI-1640 (Life Technologies) supplemented with 10% fetal bovine serum (FBS; Corning) and 1× penicillin‒streptomycin (Gibco). HFF-1 cells were cultured in Dulbecco’s modified Eagle’s medium (DMEM) supplemented with 10% FBS (Corning) and 1× penicillin‒streptomycin (Gibco). Primary cardiomyocytes were isolated from neonatal Sprague‒Dawley rats (Zhejiang Academy of Medical Sciences). Ventricles from the heart were sliced into approximately 1 mm sections in Hanks balanced salt solution (HBSS; Gibco). After digestion with collagenase II (0.2 mg/ml; Worthington) and trypsin (0.3 mg/ml, Gibco) for 1 h at 37 °C, cardiomyocytes were collected by centrifugation. Cells were then transferred to a 96-well plate or the biosensor chip previously coated with gelatin solution (Gibco). DMEM supplemented with 10% FBS and 1× penicillin‒streptomycin was used to maintain cardiomyocytes.

### Cardiomyocyte immunofluorescence staining and confocal microscopy

Cells isolated from neonatal rats were immunofluorescently stained with the cardiomyocyte-specific markers cardiac troponin I (cTnI) and cardiac muscle alpha-actin. Cells were fixed with 4% paraformaldehyde (Fisher Scientific) for 15 min, permeabilized with 0.1% Triton X-100 (Sigma‒Aldrich), and then blocked with 1% bovine serum albumin (BSA) for 1 h. Subsequently, the cells were incubated with mouse anti-α-actin antibody (A7811, Sigma) diluted 1:100 and rabbit anti-cTnI antibody (ab228847, Abcam) diluted 1:500 at 4 °C overnight. Then, the cells were stained with the following secondary antibodies: 1:200 diluted Alexa Fluor 488-labeled goat anti-mouse IgG (ab150113, Abcam) and 1:200 diluted Alexa Fluor 647-labeled goat anti-rabbit IgG (ab150079, Abcam). 6-Diamidino-2-phenylindole (DAPI) was used for nuclear counterstaining. Images were observed with a confocal microscope (Olympus FV3000, Japan).

### Cell viability assay

To evaluate AC0010-induced inhibition, cell viability was analyzed using the multifunctional cell-based biosensor and verified with a cell counting kit-8 (CCK8; Dojindo, Japan). NCI-H1975, A549, and HFF-1 cells were seeded into the multifunctional biosensor or 96-well plate at a density of 1 × 10^4^ cells per well. After overnight culture, cells were treated with AC0010 (Selleck, USA) at different concentrations for 48 h. For biosensor analysis, CI values were normalized to 1 when drugs were added. After 48 h of treatment, CI values dropped from 1 to different degrees. The cell viability ratio could be calculated by the following equation:

$${{{\mathrm{Viability}}}}\,{{{\mathrm{ratio}}}} = {{{\mathrm{CI}}}} \times 100\%$$

For CCK8 detection, 10 μl reagent was added to the culture medium 1 h before the analysis. After incubation at 37 °C for 1 h, the absorbance values at OD 490 nm were measured using a microplate reader (Molecular Devices, USA) to calculate the viability ratio.

### Live-dead assay

The live/dead kit was purchased from Dojindo, Japan. Cardiomyocytes were cultured in the multifunctional biosensor chip for five days and treated with AC0010 for two more days. Next, cardiomyocytes were incubated with the live/dead kit according to the manufacturer’s instructions. After removing excess dye, a confocal microscope (Olympus FV3000, Japan) was used for in situ imaging.

### Statistical analysis

Statistical analysis was performed with GraphPad Prism 6 (GraphPad Software Inc., USA). The mean value and standard deviation were calculated and presented. A *p* value of < 0.05 was considered statistically significant.

## Supplementary information

Supplemental material
