# Supplementary material for: Evaluating the efficacy and cardiotoxicity of EGFR-TKI AC0010 with a novel multifunctional biosensor
Source: Microsyst Nanoeng. 2023 May 10;9:57. doi: 10.1038/s41378-023-00493-4 (PMC10172296; doi:10.1038/s41378-023-00493-4)
Supplement: Supplementary file 1 — Supplemental material [file 41378_2023_493_MOESM1_ESM.docx]

**Evaluating the efficacy and cardiotoxicity of EGFR-TKI AC0010 with a novel multifunctional biosensor**

Deming Jiang^1,2^, Yong Qiu^1,2^, Yuxuan Zhu^1,2^, Xin Liu^1,2^, Fengheng Li^1,2^, Liubin Kong^1,2^, Yuxiang Pan^4^*, Hao Wan^1,2^*, Ping Wang^1,2,3^*

^1^Biosensor National Special Laboratory, Key Laboratory for Biomedical Engineering of Education Ministry, Department of Biomedical Engineering, Zhejiang University, Hangzhou 310027, China

^2^Cancer Center, Zhejiang University, Hangzhou, 310058, China

^3^State Key Laboratory for Sensor Technology, Chinese Academy of Sciences, Shanghai 200050, China

^4^Research center of smart sensing, Zhejiang lab, Hangzhou, China


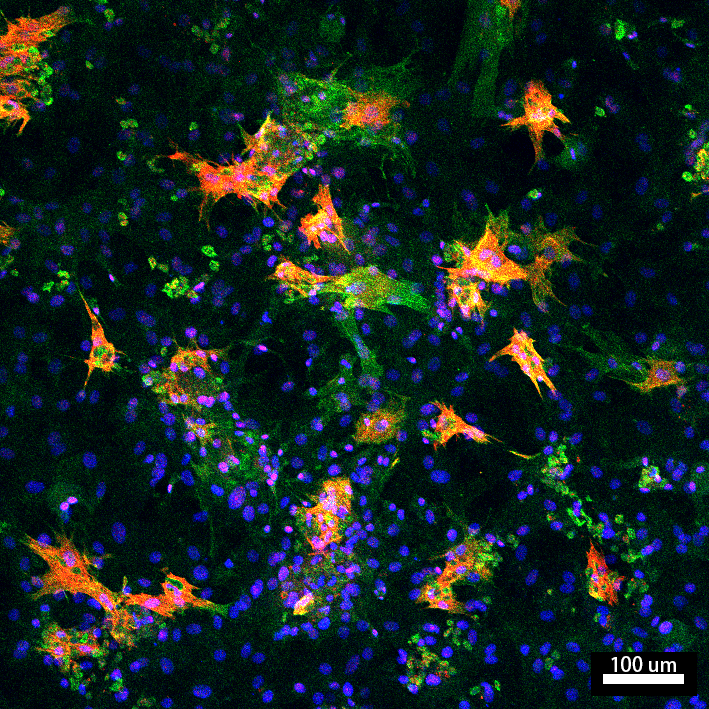


Figure S1. *Immunofluorescence image of cardiomyocytes (blue: nucleus; green: α-actin; red: cTnI).*


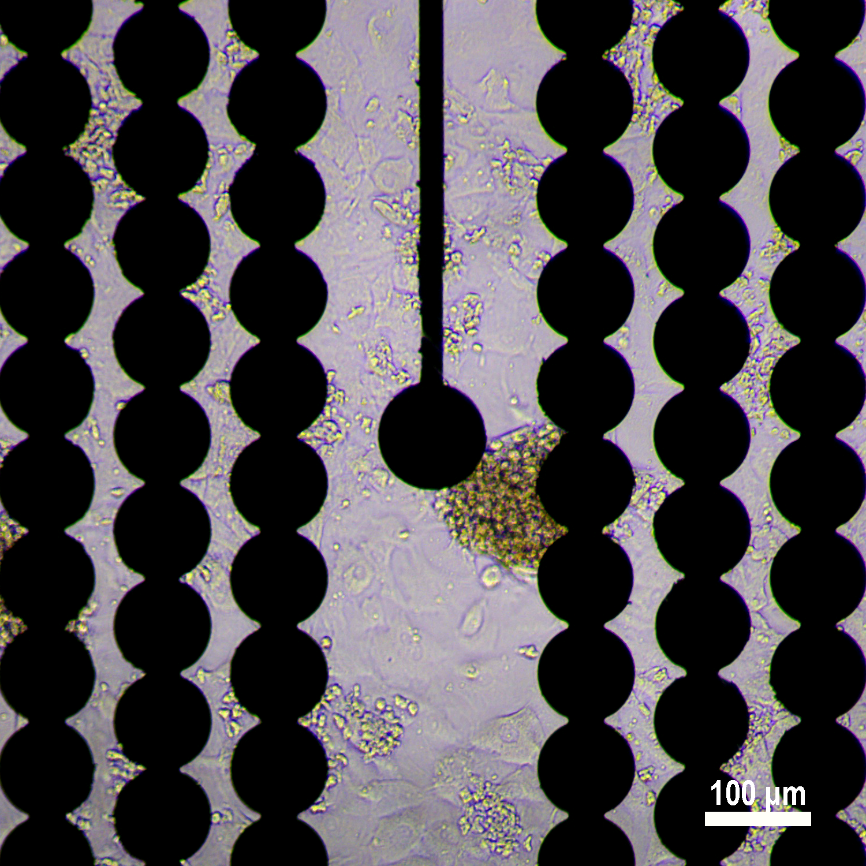


*Figure S2. Image of cardiomyocytes adhering on the electrodes.*
